# The Pathological Fear of COVID-19 and Neuroparasitosis: A Case Report of Neurocysticercosis

**DOI:** 10.7759/cureus.77655

**Published:** 2025-01-19

**Authors:** Sabina Azevedo, Sara Pereira, Rita Vilar da Mota, Carlos Gonçalves, Liliana Costa

**Affiliations:** 1 Internal Medicine - Medicina 2, Unidade Local de Saúde do Alto Minho, Ponte de Lima, PRT; 2 Internal Medicine - Medicina 1, Unidade Local de Saúde do Alto Minho, Viana do Castelo, PRT

**Keywords:** central nervous system infection, clinical anxiety, multiple parenchymal neurocysticercosis, neurocysticercosis, parasitic disease, psychiatric disease, taenia solium

## Abstract

Neurocysticercosis (NCC) is an infection of the central nervous system by the helminth *Taenia solium* (*T. solium*). The eggs of this parasite are transmitted through the faecal-oral route and by eating contaminated pork. It is currently endemic in many underdeveloped countries and is one of the main treatable risk factors for seizures and epilepsy worldwide. The clinical presentation depends on the location, number and size of the cysts. Neurological symptoms such as headache, epileptic seizures or hydrocephalus are the most common, although neuropsychiatric manifestations associated with this pathology are increasingly being recognised. Diagnosis is based on neuroimaging and serological tests. First-line treatment consists of antiparasitic drugs, corticotherapy and any necessary supportive therapy, such as anti-seizure medication. Despite the availability of treatment worldwide, NCC has been difficult to treat and radiate because its diagnosis depends on ancillary tools that are not widely available in the vast majority of countries where the disease is endemic. As a result, its prevalence is increasing in developed countries, particularly in Europe, with travel and immigration being one of the main factors. We present the case of an 82-year-old patient with a history of psychiatric disorders, who reported episodes of transient dysarthria associated with recent behavioral changes, including periods of anxiety and panic attacks due to fear of contracting COVID-19. Following initial evaluation and complementary examinations, the patient was diagnosed with active NCC. The patient underwent treatment and was discharged asymptomatic, with resolution of the anxiety and panic episodes, even in the context of a COVID-19 infection that occurred during hospitalisation. Although NCC is the most widespread parasitic central nervous system (CNS) disease worldwide, its psychiatric manifestations do not appear to be well-researched in the literature. The aim of this report is to emphasise the inclusion of NCC in the differential diagnosis not only of epileptic seizures and focal neurological deficits but also of behavioral changes.

## Introduction

*Taenia solium* is a larval cyst of the tapeworm that affects a significant number of patients around the world [1-3]. Its life cycle and reproduction depend on an intermediate host, the pig, and a definitive host, the human being. *T. solium* eggs infect people and pigs through the faecal-oral transmission or by drinking contaminated water. In turn, eating pork or accidental transmission leads to cysticercosis, a condition characterised by the formation of cysts in various tissues, including the muscle, eyes, skin and brain. The cysts that form in the brain cause neurocysticercosis (NCC), which is the most common and widespread neuroparasitosis in the world, affecting around 50 million people [2].

NCC is endemic in several areas of the world, especially those associated with worse sanitary conditions, such as Latin America, sub-Saharan Africa and Southeast Asia [1,4,5]. The lack of sanitary conditions, the proximity of people in the same dwelling and the close contact with pigs contribute to the high transmission rate and consequent infection of the population [1]. This pathology can present with intra- or extra-parenchymal lesions, and their location has an impact on the symptoms, treatment and prognosis of the disease [1,3,5]. Neurological symptoms are the most common, with a wide range of presentations from headaches to epileptic seizures or intracranial hypertension with hydrocephalus [6].

The diagnosis of NCC rests on neuroimaging tools and is supported by immunodiagnostic tests [3]. Pathological changes are reflected in magnetic resonance imaging (MRI) and, to a lesser extent, in computed tomography (CT) [7]. Symptomatic NCC requires medical therapy with symptomatic medication and/or antiparasitic drugs and, less frequently, surgical interventions [3]. Preventing calcifications in those individuals who receive antiparasitic treatment for viable disease would potentially decrease the risk of seizure relapse in a great number of individuals [7]. Since a definitive diagnosis of NCC cannot be established without the evidence provided by neuroimaging [3], diagnosing this condition presents a significant challenge in less developed countries, thereby contributing to the low treatment rates in these regions and evolution to calcification phase

Below, we present a clinical case of a patient with NCC whose initial presentation was non-neurological, in order to highlight the need to consider this pathology as a differential diagnosis in patients with psychiatric alterations.

## Case presentation

An 82-year-old male with a history of severe anxiety and depressive disorders lasting several years, including hospitalization in a psychiatric ward following his return from a war mission in Angola. In recent months, there has been a worsening of anxiety symptoms, including descriptions of panic attacks during consultations with his attending physician, who diagnosed him with anxiety disorder related to a "fear of COVID." The patient had no other significant medical history or medication.

**Figure 1:**
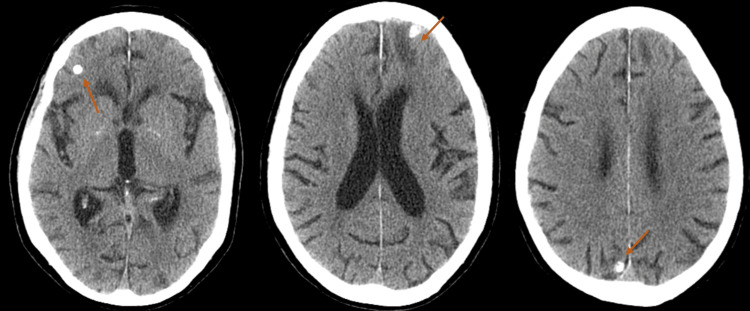
Computed tomography images with calcified lesions, the middle lesion with perilesional oedema

He was admitted to the emergency department following several episodes of transient dysarthria and left arm hypoesthesia, each lasting a few minutes, along with some behavioural changes characterized by aggressive behaviour. Upon examination in the emergency room, he exhibited mild dysarthria with no other focal neurological deficits. Laboratory tests showed elevated inflammatory markers (C-reactive protein (CRP) 18 mg/dL), and a cranial CT scan revealed a calcified lesion in the left frontal region with surrounding hypodensity, suggestive of perilesional oedema, along with other cortical calcifications without signs of activity (Figure 1).

He was admitted for further etiological investigation and dexamethasone, and anticonvulsant therapy, with levetiracetam, was initiated due to suspected cerebral oedema and potential seizure equivalents.

**Figure 2:**
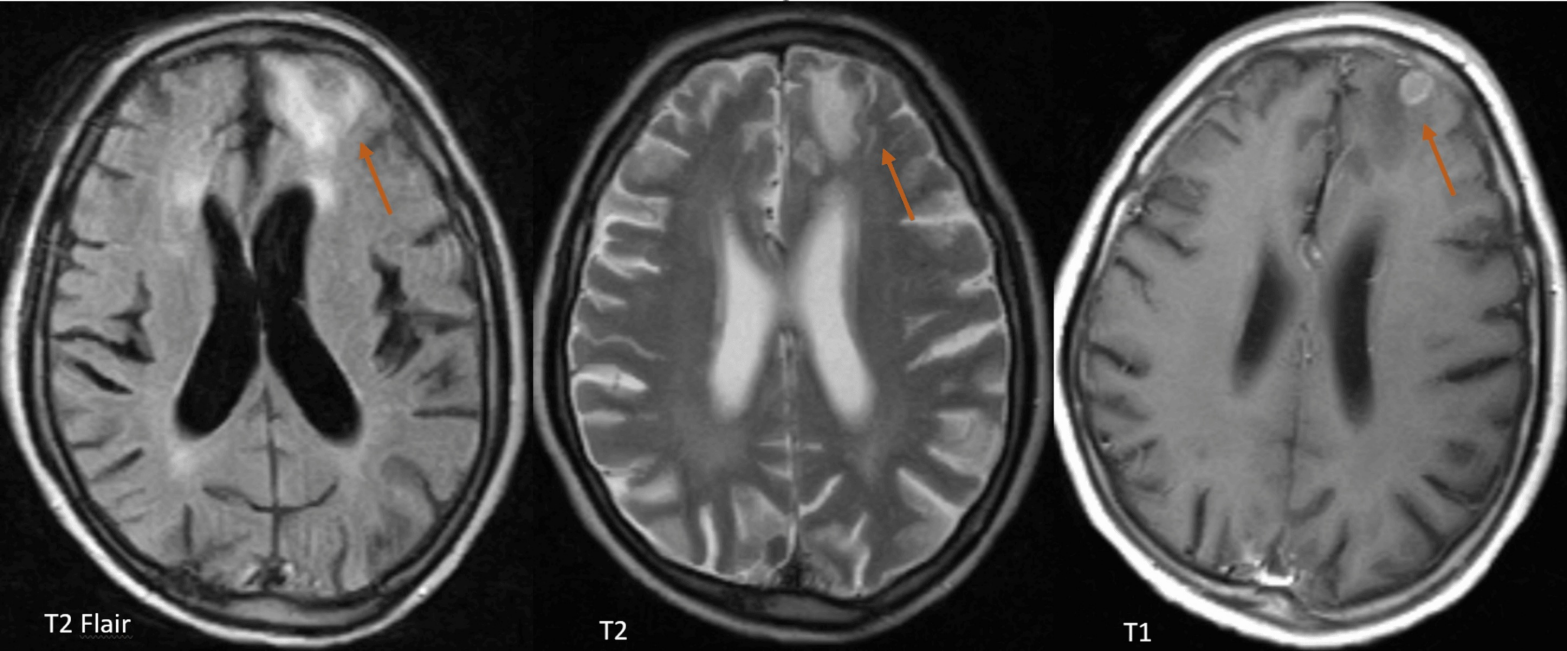
Magnetic resonance images of frontal neurocysticercosis lesions with perilesional oedema characteristic of the acute phase of the disease.

During hospitalisation, the presence of factors contributing to immunodeficiency was excluded, in particular leukopenia, hepatitis virus infections or human immunodeficiency virus. Cranial magnetic resonance imaging was performed and showed findings consistent with active-phase NCC (Figure 2).

After consultation with the infectious diseases department, *T. solium *serologies were obtained, and the diagnosis of NCC was established. Treatment was started with albendazole and therapy with dexamethasone was maintained for 20 days. Since it was not possible to perform an electroencephalogram during the hospitalization, and the episodes of dysarthria could not be excluded as epileptic equivalents, the treatment with levetiracetam was continued.

**Figure 3:**
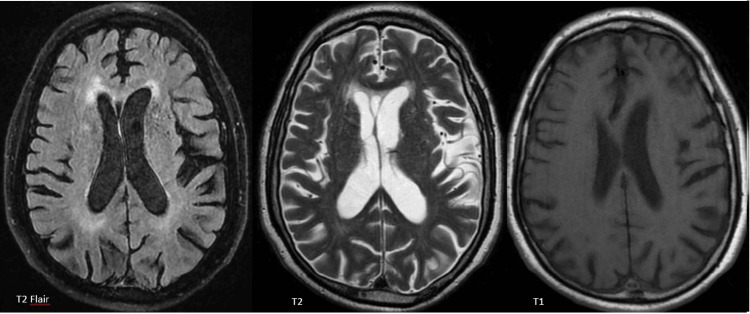
Re-evaluation magnetic resonance images at six months with resolution of previous lesions

Throughout the hospitalisation, there were no further episodes of dysarthria, neurological symptoms or comportamental changes, and the patient even contracted COVID-19 infection without behavioural changes, panic attacks or anxiety symptoms. The patient remained asymptomatic throughout hospitalisation and was discharged after completion of the therapeutic regimen. The patient was re-evaluated six months later, remaining asymptomatic with no neurological or neuropsychiatric symptoms. An MRI revealed calcified lesions with no adjacent oedema and a reduction in their size (Figure 3).

## Discussion

The association between human *T. solium* infection and neurological pathology has been well-documented since the 16th century [3]. As noted above, neurological manifestations depend not only on the location of the lesion but also on its stage of development. Four stages have been identified: the vesicular phase, the first stage during which patients typically remain asymptomatic; the colloidal phase, characterised by cyst degeneration and increased inflammatory response with consequent perilesional oedema; and finally the granular phase, followed by the cyst calcification phase. The onset or worsening of clinical manifestations is primarily associated with perilesional oedema and occurs in later stages [7-9]. In terms of location, parenchymal lesions are primarily associated with epileptic seizures and headache, whereas extraparenchymal lesions correlate with compressive symptoms such as intracranial hypertension and hydrocephalus [3,5]. Among the wide range of possible neurological manifestations, seizures are the most common, occurring in 70-90% of patients, making NCC the most common treatable cause of epilepsy worldwide [9].

In addition to the neurological changes traditionally associated with this pathology, an increasing number of concurrent psychiatric manifestations are being described. Approximately 15% of patients with NCC present with psychotic symptoms such as confusion, suicidal ideation, psychomotor agitation, aggressive behaviour or hallucinations, especially in cases with parenchymal lesions. In addition to the above symptoms, although less common, episodes of depression, anxiety or personality changes have been reported, as described in the present case [5].

NCC is often observed in immunocompromised patients, especially those with HIV infection [10]. This was not the case in the patient described above, who showed no evidence of immunocompromise during the study. Given the patient's history of living in less developed countries, it is likely that the acquisition of this parasite occurred during this period.

In fact, although endemic in many countries, it is a relatively rare disease in developed countries, with the result that medical teams are generally undertrained in its suspicion and diagnosis. However, with increased travel to countries where NCC is endemic and the growing wave of immigration, this pathology has increasingly become a pressing health concern in many developed countries, such as Europe and the United States of America [3,11].

Diagnosis is based on clinical history, characteristic signs and symptoms, imaging findings and serological testing [12]. Neuroimaging, using CT or MRI, is the most sensitive method and is therefore currently considered the gold standard for diagnosis. On the other hand, serological tests, which are widely used because of their availability and ease of collection, can give false-negative results at certain stages of the disease; therefore, their negativity does not exclude the diagnosis, as in the case presented [13]. The lumbar puncture can also play a role in the diagnosis of this pathology through the identification of specific antibodies [14].

The treatment of NCC is based on the clinical presentation and the characteristics of the lesions, particularly their number or location [4,15]. Antiparasitic drugs, albendazole or praziquantel, are the first line of treatment [1-15], is recommended to administer albendazole alone for one to two viable lesions and albendazole plus praziquantel for three or more viable lesions [7]. The recommended dose of albendazole is 15 and 30 mg/kg/day, to be taken in two doses per day, for eight to 10 days. Furthermore, 15 mg/kg/day is sufficient for parenchymal NC, while in the case of extraparenchymal disease, 30 mg/kg/day has shown to be more efficient. The recommended dose of praziquantel is 50 mg/kg/day for 10 to 15 days [4]. The use of corticosteroids is associated with a reduction in inflammatory activity and should be given before antiparasitic drugs [15]; commonly used doses include prednisone 1 mg/kg/day or dexamethasone 0.1 mg/kg/day, begun one day prior to antiparasitic therapy, continued for the duration of antiparasitic therapy and followed by a rapid taper [8]. In patients with epileptic seizures, antiepileptic drugs should be administered [15]. Surgical intervention can be a therapeutic weapon, especially to relieve hydrocephalus [15,16]. Follow-up imaging can be done every six months until the lesions resolve [12].

## Conclusions

NCC is highly prevalent in developing countries, being the leading cause of seizures in young people in many of these nations. However, its prevalence is low in Europe, with most physicians being unfamiliar with its management. Diagnosis can be particularly challenging in cases where there is no clear history of previous exposure to *T. solium*. Therefore, in patients presenting with neurological symptoms and evidence of intra- or extracranial lesions, a thorough clinical history is essential, particularly regarding raw pork consumption or travel to high-risk countries.

This case aims to highlight this pathology and the necessity of its consideration in cases of intracerebral lesions, even in non-endemic areas. Furthermore, it intends to emphasize that beyond the widely described neurological symptoms of this pathology, it is also important to be vigilant for psychiatric symptoms such as anxiety, depression, aggressive behaviour or even hallucinations, which, although less documented, can be manifestations of NCC, as demonstrated in the described case. Thus, the primary objective is to bring attention to this pathology, emphasizing its clinical presentation and the importance of clinical suspicion, so that its recognition is timely and allows for early initiation of treatment, which is crucial for the prevention of adverse outcomes.
